# Covalent Immobilization of *Bacillus licheniformis* γ-Glutamyl Transpeptidase on Aldehyde-Functionalized Magnetic Nanoparticles

**DOI:** 10.3390/ijms14034613

**Published:** 2013-02-26

**Authors:** Yi-Yu Chen, Ming-Gen Tsai, Meng-Chun Chi, Tzu-Fan Wang, Long-Liu Lin

**Affiliations:** 1 Department of Applied Chemistry, National Chiayi University, 300 Syuefu Road, Chiayi City 60004, Taiwan; E-Mails: s0972236@mail.ncyu.edu.tw (Y.-Y.C.); s0992739@mail.ncyu.edu.tw (M.-G.T.); s0910324@mail.ncyu.edu.tw (M.-C.C.); 2 Department of Life Sciences and Institute of Molecular Biology, National Chung Cheng University, Chiayi County 621, Taiwan

**Keywords:** *Bacillus licheniformis*, γ-glutamyl transpeptidase, magnetic nanoparticle, 3-aminopropyltriethoxysilane, covalent immobilization

## Abstract

This work presents the synthesis and use of surface-modified iron oxide nanoparticles for the covalent immobilization of *Bacillus licheniformis* γ-glutamyl transpeptidase (*Bl*GGT). Magnetic nanoparticles were prepared by an alkaline solution of divalent and trivalent iron ions, and they were subsequently treated with 3-aminopropyltriethoxysilane (APES) to obtain the aminosilane-coated nanoparticles. The functional group on the particle surface and the amino group of *Bl*GGT was then cross-linked using glutaraldehyde as the coupling reagent. The loading capacity of the prepared nanoparticles for *Bl*GGT was 34.2 mg/g support, corresponding to 52.4% recovery of the initial activity. Monographs of transmission electron microscopy revealed that the synthesized nanoparticles had a mean diameter of 15.1 ± 3.7 nm, and the covalent cross-linking of the enzyme did not significantly change their particle size. Fourier transform infrared spectroscopy confirmed the immobilization of *Bl*GGT on the magnetic nanoparticles. The chemical and kinetic behaviors of immobilized *Bl*GGT are mostly consistent with those of the free enzyme. The immobilized enzyme could be recycled ten times with 36.2% retention of the initial activity and had a comparable stability respective to free enzyme during the storage period of 30 days. Collectively, the straightforward synthesis of aldehyde-functionalized nanoparticles and the efficiency of enzyme immobilization offer wide perspectives for the practical use of surface-bound *Bl*GGT.

## 1. Introduction

Enzymes are versatile biocatalysts that are useful for biotechnological applications, including food industries and pharmaceuticals [1,2]. The specific chemo-, regio- and stereo-selectivity of enzyme-mediated transformations remarkably render their superiority over chemical catalytic reactions [3–5]. For industrial applications, immobilized enzymes are currently the object of considerable interest, because of the advantage benefits over the soluble form of enzymes or alternative technologies [6]. The ability to retain or recover enzymes allows biocatalyst separation from the product, thereby permitting continuous processes, and prevents carry-through of protein or activity to subsequent process steps [7]. In some cases, the denaturation operation of immobilized enzymes can even be prevented [8–10]. Although the obvious advantages of enzyme immobilization have been reported [6,11], it is estimated that only 20% of the biological processes involve the utilization of immobilized enzymes [12]. However, a number of interesting findings indicate that enzyme immobilization has entered an exciting new phase [13].

The selected approach of immobilized enzymes should be able to stabilize the target biocatalysts and allow easier diffusion of substrates and products. Stabilization of enzymes against many different types of inactivation has been accomplished by a variety of immobilization strategies, including adsorption, polymer entrapment, microencapsulation, chemical aggregation, bioaffinity and covalent coupling [6,11,13]. Among the immobilization methods employed, covalent cross-linking of enzymes to inert supports is practically useful, since it can facilitate product separation and sometimes helps to improve the stability of biocatalysts [14–18]. Nanomaterials are currently attracting much attention as ideal supports for enzyme immobilization, since they could provide a very high-specific area surface for enzyme loading, lower the limitation of substrate and product diffusion and improve the catalytic efficiency [19]. To date, a variety of nanomaterials, including carbon nanotube, epoxy nanobeads and cellulose-bound magnetic nanoparticles, have been fabricated and used for the covalent immobilization of enzymes [20–22].

γ-Glutamyl transpeptidase (GGT) belongs to the N-terminal nucleophile (Ntn) hydrolase superfamily [23] and catalyzes the transfer of the γ-glutamyl moiety of glutathione to an amino acid, a short peptide or water molecule [24]. The gene encoding GGT is translated as a unique polypeptide that then undergoes an autocatalytic processing to form either a heterodimeric or heterotetrameric enzyme comprising of the typical large (L) and small (S) subunits [25–27]. The mature GGT enzyme plays a key role in the γ-glutamyl cycle, a pathway for the biosynthesis and degradation of glutathione, as well as xenobiotic detoxification [28–30]. Beyond its physiological function, GGT enzymes can be employed for the synthesis of γ-glutamyl derivatives, with great potential for pharmaceutical applications [31].

**Figure 1:**
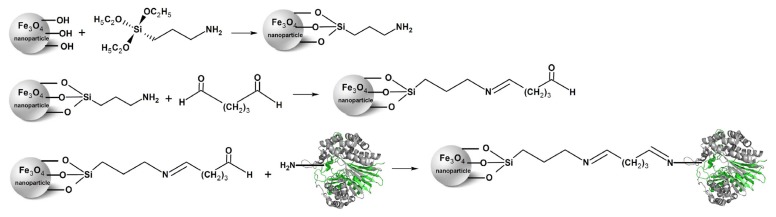
The strategy used to immobilize *Bacillus licheniformis* γ-glutamyl transpeptidase (*Bl*GGT) on the magnetic nanoparticles.

The putative *B. licheniformis ggt* gene can be translated into a 61.259 kDa precursor consisting of a signal peptide of 25 residues, a L-subunit of 374 residues and a S-subunit of 187 residues (Swiss-Prot Q65KZ6). Earlier, a recombinant *B. licheniformis* GGT (*Bl*GGT) was functionally expressed in *Escherichia coli* M15 cells [32]. Deletion analyses of the recombinant enzyme have demonstrated that both N- and C-terminal sequences are crucial for the expression of its active form in host cells [33,34]. Given that nano-sized magnetic particles have recently received considerable attention for chemical cross-linking of enzymes [22,34–38], the use of surface-modified magnetic nanoparticles to covalently immobilize *Bl*GGT should be a persuasive approach for obtaining stable and reusable biocatalyst preparations. In this work, we coated the prepared Fe_3_O_4_ nanoparticles with aminosilane and, subsequently, immobilized *Bl*GGT on the nanomaterial through a glutaraldehyde coupling reaction (Figure 1). Moreover, the chemical properties and kinetic parameters of the immobilized *Bl*GGT were determined and compared with its soluble counterpart.

## 2. Results and Discussion

### 2.1. Immobilization of the Recombinant Enzyme

In the present study, the purified *Bl*GGT was covalently attached to the surface-modified magnetic nanoparticles. In order to obtain an immobilized biocatalyst with a high amount of enzyme loading and activity recovery, the effects of initial enzyme concentration and coupling time on the immobilization efficiency were investigated.

**Figure 2:**
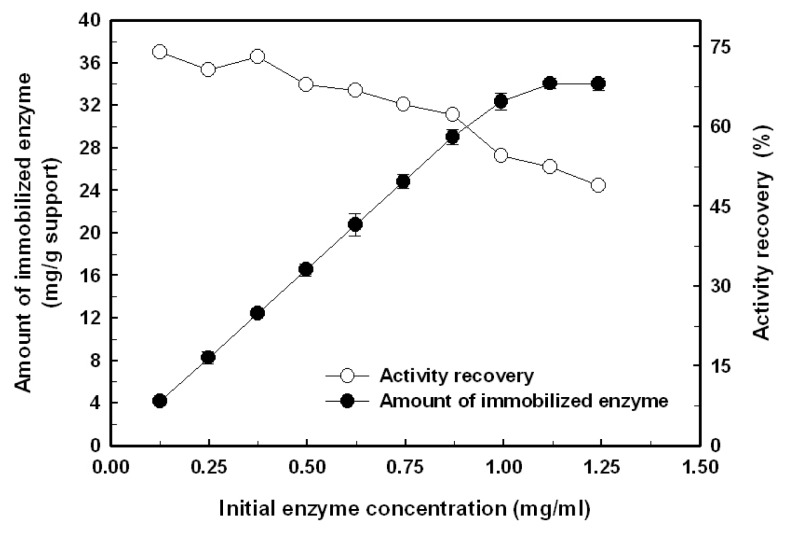
Effect of initial *Bl*GGT concentration on the amount of immobilized enzyme (closed circles) and activity recovery (open circles).

The effect of the initial *Bl*GGT concentration on the amount of the biocatalyst covalently attached on the surface-modified magnetic nanoparticles and recovery of the enzymatic activity are shown in Figure 2. The amount of immobilized enzyme almost proportionally increased with the increment of initial enzyme concentration in the bulk solution from 0.125 to 0.875 mg/mL and then leveled off. Eventually, the maximum loading for *Bl*GGT could reach 34.2 mg/g of support, which corresponds to an activity recovery of 52.4%. The activity recovery of *Bl*GGT, defined as the ratio of the GGT activity of immobilized enzyme to the initial activity, was almost kept unchanged in the low range of initial enzyme concentration (0.125–0.375 mg/mL). However, a further increase in the initial enzyme concentration led to the reduction of activity recovery. The possible explanation for this phenomenon is that the multiple layers of enzyme molecules on the surface of magnetic nanoparticles are formed at high enzyme loading, which might block the active sites of the enzyme and cause the diffusion limitation of the substrate.

**Figure 3:**
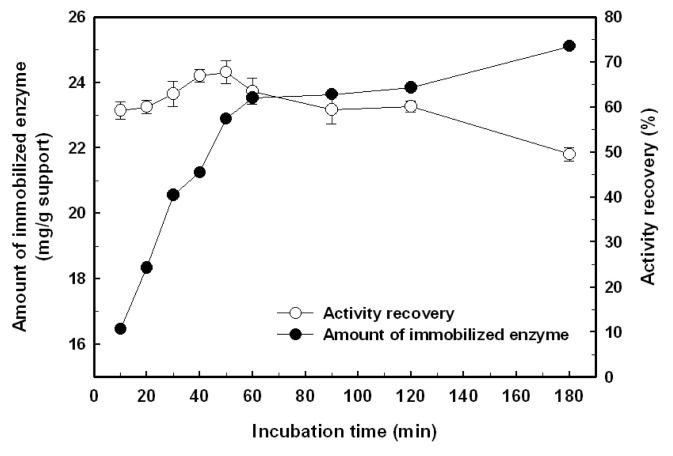
Effect of the coupling time on the amount of immobilized *Bl*GGT (closed circles) and activity recovery (open circles). In this experiment, the initial enzyme concentration was 0.92 mg/mL.

The effect of coupling time on the immobilization efficiency of *Bl*GGT on the 3-aminopropyltriethoxysilane (APES)-modified magnetic nanoparticles is presented in Figure 3. The amount of immobilized enzyme was increased with the increment of coupling time, reaching a plateau after 1 h and the maximum amount of enzyme immobilization was determined to be 25.1 mg/g of support. The activity recovery of immobilized *Bl*GGT maintained almost constant at about 57.7% for the first 50 min and then gradually decreased when the incubation time went further. A long immobilization time resulted in lower activity recovery, which could be the result of the creation of additional multiple covalent linkages between the nanoparticles and the enzyme, and, in turn, disturbs the structural conformation of the biocatalyst.

### 2.2. Properties of the Magnetic Nanoparticles

**Figure 4:**
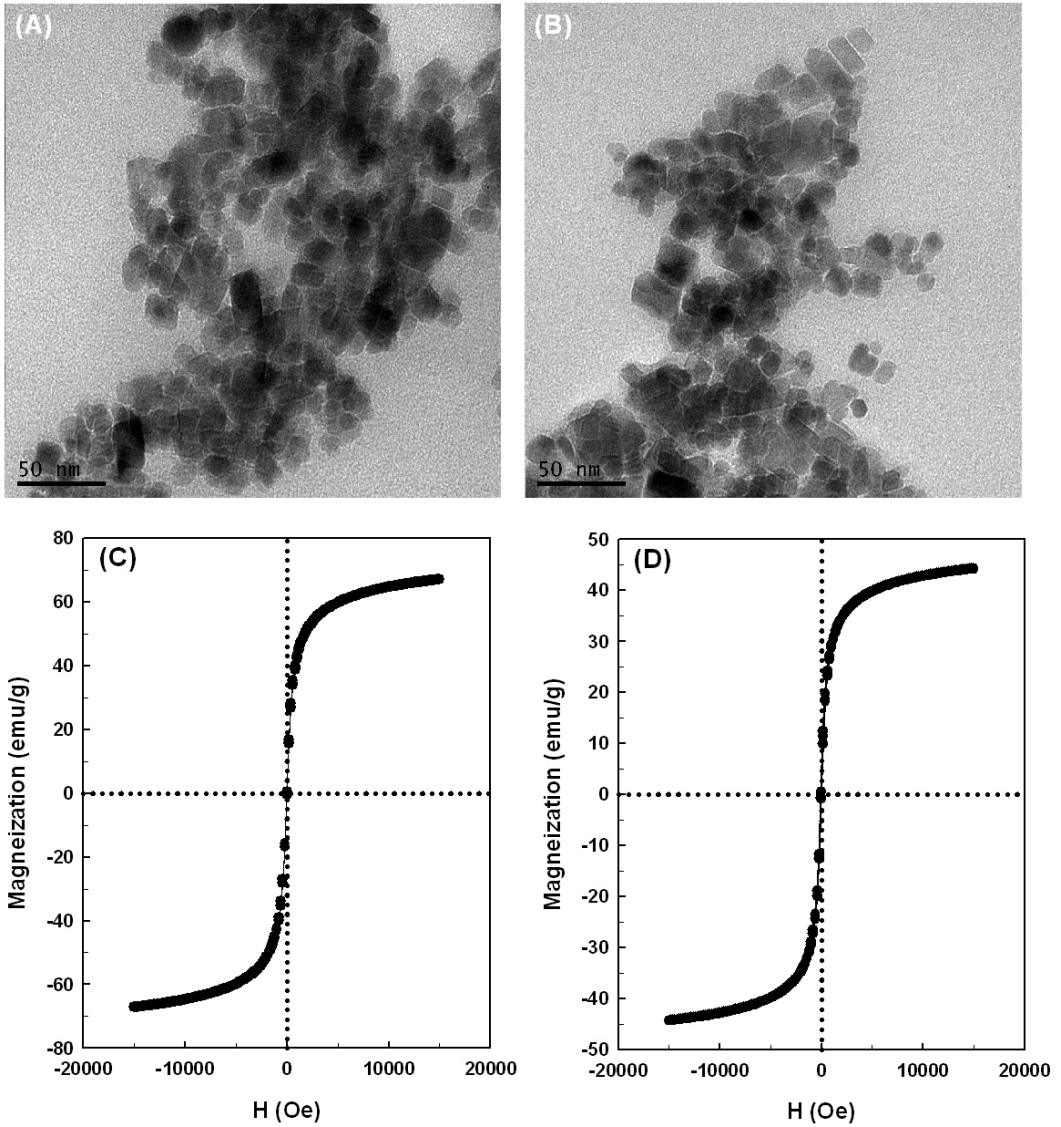
Transmission electron microscopy (TEM) micrographs (**A**) and (**B**) and magnetization curves (**C**) and (**D**) of Fe_3_O_4_ and Fe_3_O_4_/APES/*Bl*GGT. Panels represent: (**A**) and (**C**), naked Fe_3_O_4_; (**B**) and (**D**), Fe_3_O_4_/3-aminopropyltriethoxysilane (APES)/*Bl*GGT.

Typical transmission electron microscopy (TEM) micrographs for the magnetic nanoparticles without and with covalently cross-linked *Bl*GGT are shown in Figure 4. The magnetic nanoparticles had a size distribution of 7.5–25.3 nm, and the statistical sampling of 103 particles in five TEM micrographs yielded a mean diameter of 15.1 ± 3.7 nm (Figure 4A). The particle diameter is believed to be an important feature for the immobilization matrix. Definitely, smaller particles have larger surface-to-volume ratios and larger capacity to bind more enzymes on their surface, and the substrate and product would give less restriction for diffusion [39,40]. In this case, the particles remained discrete after cross-linking to *Bl*GGT and had a mean diameter of 17.2 ± 3.9 nm (Figure 4B), which is comparable to that of uncross-linked ones. These observations reveal that the immobilization step does not significantly change the average size of the magnetic nanoparticles.

The magnetization curves of naked Fe_3_O_4_ and Fe_3_O_4_/APES/*Bl*GGT measured at 300 K showed no detectable coercivity in the field sweep (Figure 4C,D), which suggests that the magnetic nanoparticles possess a characteristic of superparamagnetic materials [41], and the saturation magnetization of Fe_3_O_4_ and Fe_3_O_4_/APES/*Bl*GGT was different with individual values of 67.2 and 42.1 emu/g, respectively. This indicates that the magnetic properties of Fe_3_O_4_/APES/*Bl*GGT have a content of 62.6% with respect to the value of naked Fe_3_O_4_. Besides, the enzyme-conjugated magnetic nanoparticles in aqueous solution were a brown suspension, and they were easily recovered from the solution under an external magnetic field (data not shown). Once the external magnetic field was removed, they could be redispersed into a particle suspension with slight shaking. Based on the magnetization curves and the separation-redispersion process, it can be concluded that the prepared nanoparticles have the superparamagnetic properties.

**Figure 5:**
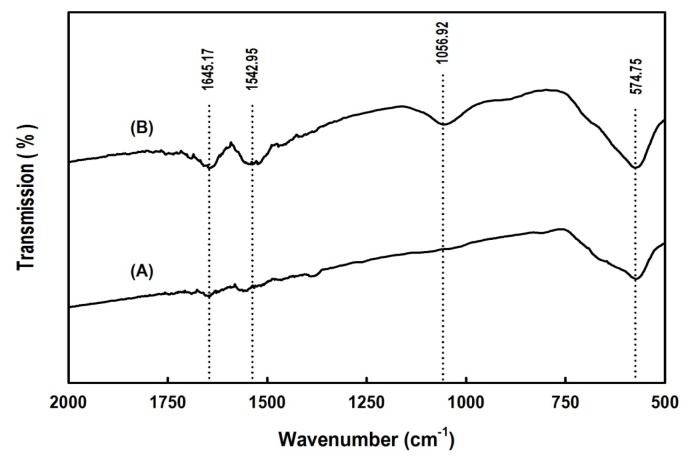
Fourier transform infrared spectroscopy (FTIR) spectra of naked Fe_3_O_4_ and Fe_3_O_4_/APES/*Bl*GGT. Lines represent: (**A**) naked Fe_3_O_4_; (**B**) Fe_3_O_4_/APES/*Bl*GGT.

The cross-linking of *Bl*GGT to the aldehyde-functionalized magnetic nanoparticles was also confirmed by FTIR analysis. Figure 5 shows the FTIR spectra of naked Fe_3_O_4_ and Fe_3_O_4_/APES/*Bl*GGT. The peak at 569 cm^−1^ relates to the Fe–O group of Fe_3_O_4_. Compared with the spectrum of naked Fe_3_O_4_, the characteristic bands appear at 1542.95 and 1645.17 cm^−1^ in Fe_3_O_4_/APES/*Bl*GGT and can be assigned to the NH and NH_2_ bending vibration, respectively. These results indicate that the *Bl*GGT molecules are conjugated with the APES-modified magnetic nanoparticles successfully.

### 2.3. Characterization of Free and Immobilized Enzymes

**Figure 6:**
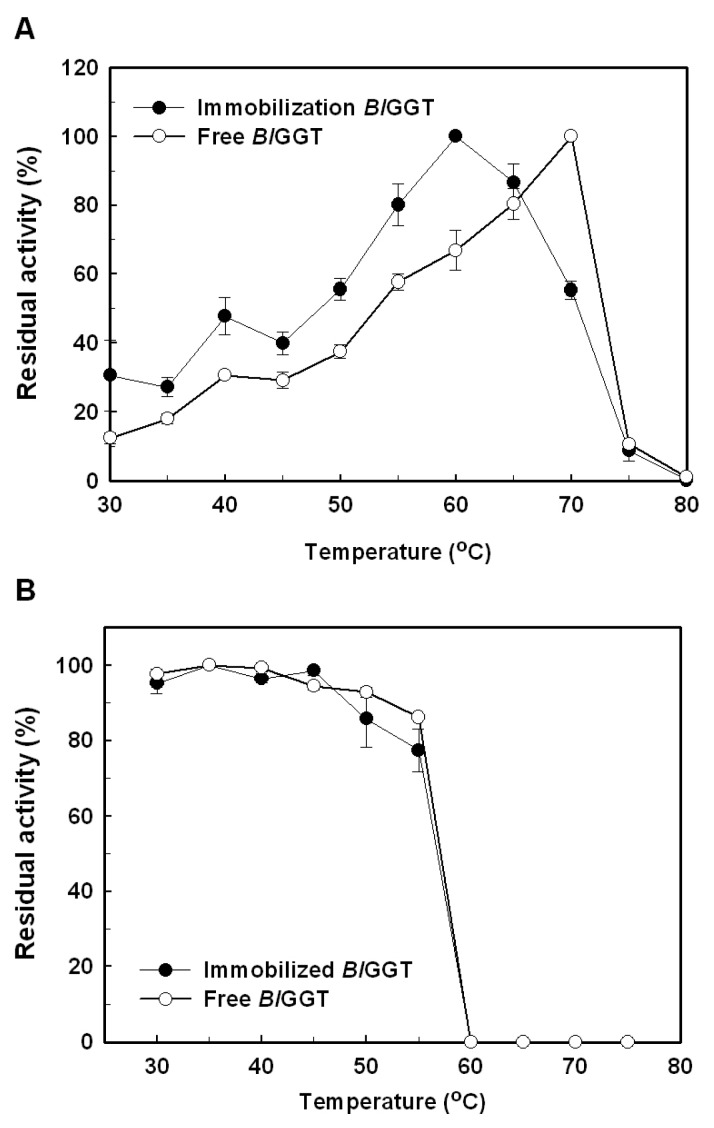
Effect of temperature on activity (**A**) and stability (**B**) of free and immobilized enzymes. Results were reported as means of three independent experiments, and the standard deviations were lower than ±4.3%.

The optimal temperature for free *Bl*GGT was seen at 70 °C, but the maximal activity of immobilized enzyme was shifted to 60 °C (Figure 6A). The thermal stability of free and immobilized *Bl*GGTs was further determined by incubating the enzyme samples in a temperature range of 30–80 °C and, thereafter, measuring their residual activity. As shown in Figure 6B, both free and immobilized enzymes shared a similar profile of thermal stability. Surface immobilization of an enzyme on magnetic particles has been shown to improve the thermal stability of the biocatalyst, probably due to the increase of its molecular rigidity [42]. Thermal stabilization by immobilization could also be observed in a number of enzymes conjugated with magnetic nanoparticles [43–45]. However, the reasons for a downshift of the optimal temperature and no significant thermostabilization of *Bl*GGT by the immobilization remain to be elucidated.

**Figure 7:**
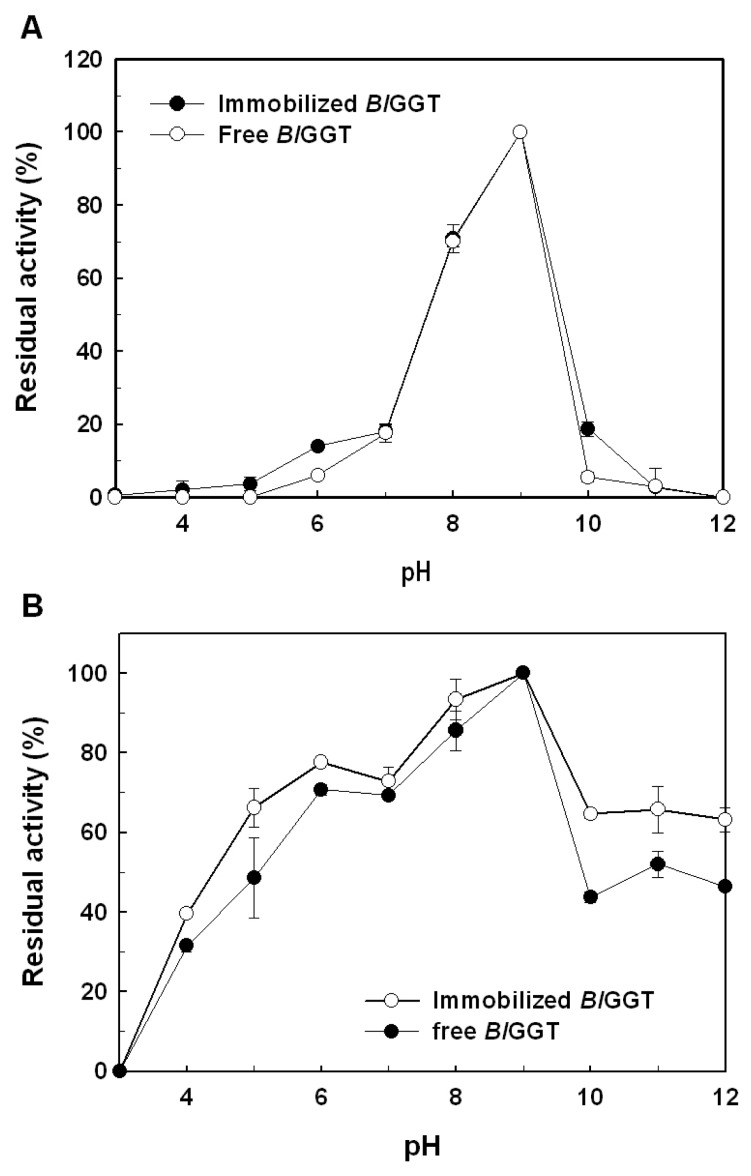
Effect of pH on activity (**A**) and stability (**B**) of free and immobilized enzymes. Results were reported as means of three independent experiments, and the standard deviations were lower than ±3.7%.

The effect of pH on the enzymatic activity of immobilized *Bl*GGT is shown in Figure 7. The optimal pH of immobilized *Bl*GGT was observed at 8.0, which is almost the same with respect to free enzyme (Figure 7A). A higher stability for the immobilized *Bl*GGT was kept in the pH range of 6–9, and the free enzyme was also in the same pH range (Figure 7B). Practically, it would be ideal if the immobilized enzyme could be functional over a wide range of pH values.

**Figure 8:**
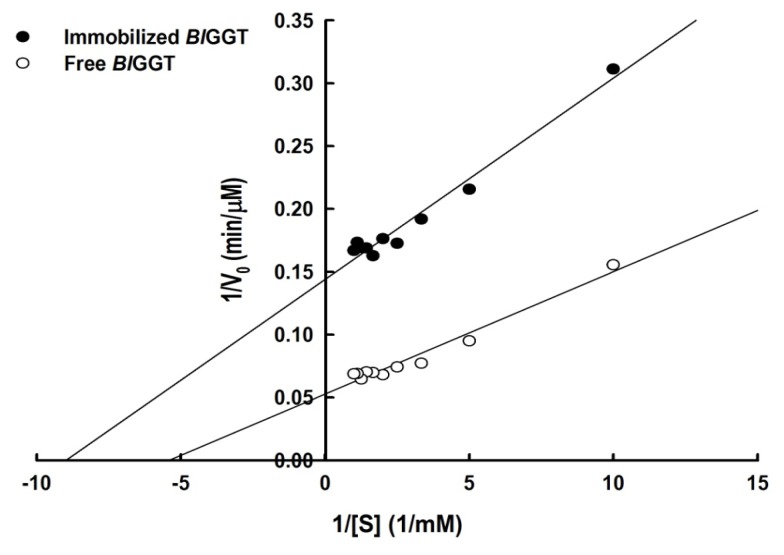
Lineweaver–Burk plot for free and immobilized enzymes.

Lineweaver-Burk plots for free and immobilized enzymes on the transpeptidation reaction of l-γ-Glu-*p*-NA were used to obtain kinetic parameters (Figure 8). The Michaelis–Menten constants, *K*_M_ and *V*_max_, for free enzyme were 0.18 mM and 18.94 μmol/min/mg, respectively. The immobilized *Bl*GGT had an apparent *K*_M_ of 0.11 mM and a *V*_max_ value of 7.01 μmol/min/mg, leading to a reduction of the catalytic efficiency (*V*_max_/*K*_M_) from 10.51 to 3.05 s^−1^. These observations indicate that the immobilization process significantly reduces the substrate affinity of *Bl*GGT and may also hinder the accessibility of reactants to the active site. These results are consistent with the kinetic observations of other enzymes immobilized on magnetic nanomaterials [37,43,46–48].

### 2.4. Reusability

**Figure 9:**
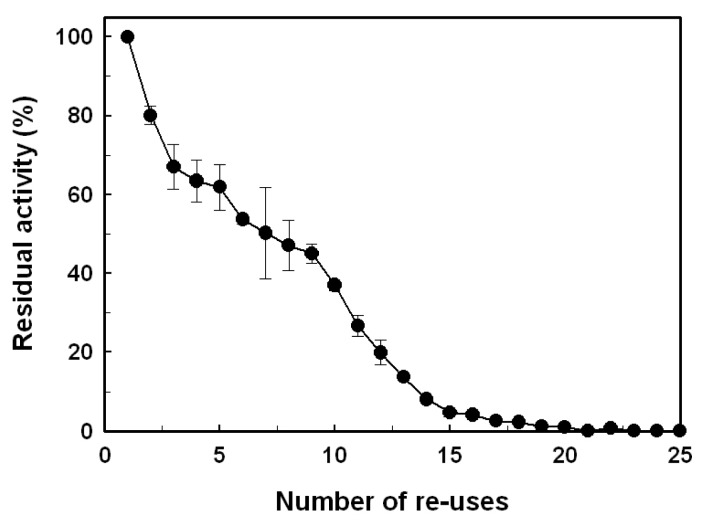
Operational stability of the immobilized enzymes. Results were reported as the means of three independent experiments, and the standard deviations were lower than ±5.2%.

The duration of a biocatalyst is an important aspect to consider its potential for industry applications. In this regard, the operational stability of immobilized *Bl*GGT was evaluated in a repeated batch process. At each of the repetitive cycles, the immobilized enzyme was recovered by magnetic separation and recycled for the transpeptidation reaction of l-γ-Glu-*p*-NA. As shown in Figure 9, the GGT activity of immobilized enzyme did not decrease significantly during the first cycle. The immobilized enzyme retained approximately 36.2% of the initial activity, even after 10 cycles of usage. Given that *Bl*GGT was stable for 24 h at 40 °C (data not shown), the gradual loss of the GGT activity may be attributed to the conformational changes of immobilized enzyme during reuse or a distributive stability of each enzyme due to differences in the number of interactions between enzyme molecules and the support matrix [49–52].

### 2.5. Storage Stability of Free and Immobilized Enzymes

**Figure 10:**
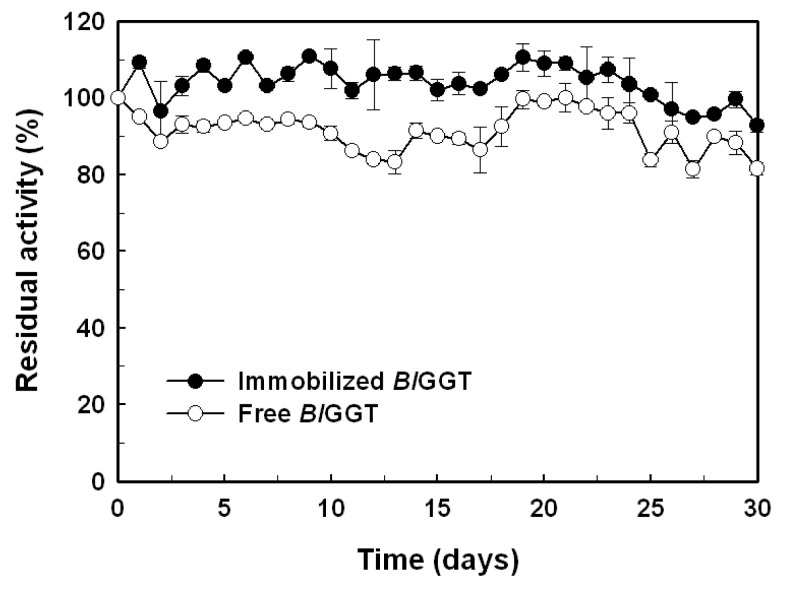
Storage stability of free and immobilized enzymes. Results were reported as means of three independent experiments, and the standard deviations were lower than ±4.1%.

The storage stability of an enzyme is important criteria for biocatalyst-mediated processes, due to the economics of industrial bioprocesses, which are closely tied to the production cost of enzymes. In this regard, we also evaluated the storage stability of free and immobilized enzymes (Figure 10). After an incubation period of 30 days, the residual activity of free and immobilized enzymes was 90.2% and 82.3%, respectively. The experimental results suggest that *Bl*GGT is quite stable after being conjugated to the surface-modified magnetic nanoparticles.

## 3. Materials and Methods

### 3.1. Chemicals

Ferric chlorides 6-hydrate, ferrous chloride tetrahydrate, glutaraldehyde and 3-aminopropyltriethoxysilane (APES) were acquired from Wako Pure Chemicals Industries, Ltd. (Osaka, Japan). Nickel-nitrilotriacetate (Ni^2+^-NTA) resin was purchased from Qiagen Inc. (Valencia, CA, USA). Chemical compounds for enzyme assay, including l-γ-glutamyl-*p*-nitroanilide (l-γ-Glu-*p*-NA), Gly-Gly and *p*-nitroaniline (*p*-NA) were brought from Sigma-Aldrich Fine Chemicals (St. Louis, MO, USA). All other chemicals were commercial products of analytical or molecular biological grade.

### 3.2. Expression and Purification of the Recombinant GGT

To purify *Bl*GGT from the crude extract of *E. coli* M15 (pQE-*Bl*GGT) [32], the recombinant bacterium was grown at 37 °C in 100 mL of Luria-Bertani (LB) medium containing 100 μg ampicillin mL^−1^ and 25 μg kanamycin mL^−1^ to an absorbance at 600 nm of 1.0. Then, isopropyl-β-D-thiogalactopyranoside (IPTG) was added to a final concentration of 0.1 mM, and the cultivation of bacteria continued at 20 °C for 12 h. Bacterial cells were harvested by centrifugation at 9000 × *g* for 10 min at 4 °C, resuspended in 30 mL of 20 mM Tris-HCl buffer (pH 8.0) after decanting the supernatant and disrupted by sonication (Sonicator XL-2000; Misonix, Inc., Farmingdale, NY, USA). The extract was clarified by centrifugation at 12,000× *g* for 20 min at 4 °C, and the resulting supernatant was mixed with Ni^2+^-NTA resin pre-equilibrated with the binding buffer (5 mM imidazole, 0.5 M NaCl and 20 mM Tris-HCl; pH 7.9). The adherent *Bl*GGT was eluted from the column by an elution buffer consisting of 0.5 M imidazole, 0.5 M NaCl and 20 mM Tris-HCl (pH 7.9).

### 3.3. Determination of GGT Activity

GGT activity was assayed at 50 °C according to the method of Orlowski and Meister [53], and the formation of *p*-NA was recorded by monitoring the absorbance changes at 410 nm. The reaction mixture contained 1.25 mM l-γ-Glu-*p*-NA, 30 mM Gly-Gly, 1 mM MgCl_2_, 20 mM Tris-HCl buffer (pH 8.0) and 20 μL of enzyme solution at a suitable dilution and enough distilled water to bring the final volume to 1 mL. One unit of GGT activity is defined as the amount of enzyme required to produce 1 μmol of *p*-NA per min under the assay conditions.

Kinetic parameters of free and immobilized enzymes were estimated by measuring *p*-NA production in 1 mL of 20 mM Tris-HCl buffer (pH 8.0) containing various concentrations of l-γ-Glu-*p*-NA (0.1–2.0 *K*_M_), 30 mM Gly-Gly, 1 mM MgCl_2_ and an appropriate amount of free and immobilized enzymes. The *K*_M_ and *V*_max_ values were calculated from the slope and the *y*-axis intercept, respectively, on the Lineweaver-Burke plot.

### 3.4. Preparation of Aldehyde-Functionalized Magnetic Nanoparticles and Enzyme Immobilization

Magnetic nanoparticles (Fe_3_O_4_) were prepared by hydrothermal co-precipitation of ferric and ferrous salts in an alkaline solution, followed by washing the precipitates with hot water [54]. Briefly, iron(II) chloride and iron(III) chloride (1:2) were dissolved in nanopure water at a concentration of 0.25 M iron ions and chemically precipitated at ambient temperature by repeatedly adding 1 M NaOH to keep a constant pH of 10.0. The precipitates were subsequently heated at 80 °C for 30 min under vigorous stirring and washed 4 times with water and several times with ethanol. During washing, the magnetic nanoparticles were separated from the washing liquid by a magnetic separator of N48M (MAG City Co., LTD., Taiwan) of 48 mega-oersteds. The nanoparticles were finally dried in a vacuum oven at 70 °C.

To prepare APES-bound magnetic nanoparticles, 0.5 g Fe_3_O_4_ was dispersed in 9.7 mL of ethanol by sonication. After adding 0.3 mL of APES, the reaction mixture was homogenized by a sonicator for 10 min, and the sample bottle was then rolled up and down overnight at 25 °C. The APES-bound magnetic nanoparticles were subsequently recovered from the reaction mixture by placing the bottle on a permanent magnet with a surface magnetization of 48 mega-oersteds. The resulting supernatant was discarded, and the precipitates were washed several times with ethanol.

The purified *Bl*GGT was essentially immobilized on APES-bound magnetic nanoparticles through glutaraldehyde linkage [38]. The binding percentage of *Bl*GGT was estimated by determining the amount of protein in the unreacted fraction. Protein concentration was determined by the colorimetric method at 595 nm with a Bio-Rad protein assay kit using bovine serum albumin as the reference standard.

### 3.5. Characterization of Magnetic Nanoparticles

The morphology and average size of the prepared nanoparticles were determined by transmission electron microscopy (TEM) using a JEM-1400 transmission electron microscope (JEOL Ltd., Tokyo, Japan). Samples for TEM analyses were prepared by placing a drop of the nanoparticle-ethanol suspension onto a Formvar covered copper grid and evaporated in air at ambient temperature.

The magnetic property was measured using Super-conducting Quantum Interference Device Magnetometer (SQUID, MPMS7, quantum design) at the temperature of 300 K and in a magnetic field up to 50 kOe.

The covalent immobilization of *Bl*GGT on the aldehyde-functionalized magnetic nanoparticles was checked using FTIR spectra with KBr discs in the range of 2000–500 cm^−1^ on a Shimadzu FTIR-8400 spectrometer (Shimadzu Corporation, Kyoto, Japan).

### 3.6. Effects of Temperature and pH

The effect of temperature on free and immobilized enzymes was evaluated by incubating free *Bl*GGT (8.7 U/mL) and *Bl*GGT-conjugated magnetic nanoparticles (34.1 mg; wet weight) in 10 mL of 20 mM Tris-HCl buffer (pH 8.0) at various temperatures (30–80 °C) for 10 min. The amount of GGT activity was determined according to the assay procedure described above. The thermal stability of free and immobilized enzymes was performed in 10 mL of 20 mM Tris-HCl buffer (pH 8.0) at different temperatures (30–80 °C). After 30 min of incubation, the residual activity was determined under the standard assay conditions. The experiments were performed in triplicate, and the data were expressed as mean values.

To investigate the pH effect on free and immobilized enzymes, *Bl*GGT (8.7 U/mL) and *Bl*GGT-conjugated magnetic nanoparticles (34.1 mg; wet weight) were incubated with 10 mL of 20 mM Tris-maleate buffer (pH 4.5–6.0), 20 mM potassium phosphate buffer (pH 6.0–8.0), 20 mM Tris-HCl buffer (pH 8.0–9.0) or 20 mM glycine-NaOH buffer (pH 9.0–11.0) at 60 °C, and the amount of GGT activity was determined according to the standard assay conditions. For the measurement of pH stability, both free and immobilized enzymes were kept at 4 °C for 30 min in different buffers. The residual GGT activity was determined under the standard assay conditions. The experiments were performed in triplicate, and the data were expressed as mean values.

### 3.7. Reusability of the Immobilized Enzyme

The immobilized *Bl*GGT was repeatedly used to catalyze the transpeptidation of l-γ-Glu-*p*-NA in the batch process. Enzyme-conjugated magnetic nanoparticles (12 mg) in 1 mL of 20 mM Tris-HCl buffer (pH 8.0) containing 1.25 mM l-γ-Glu-*p*-NA, 30 mM Gly-Gly, 1 mM MgCl_2_ and 20 mM Tris-HCl buffer (pH 8.0) were shaken (100 rpm) at 50 °C for 10 min each time. The GGT activity was immediately determined under the standard assay conditions. After each cycle of process, the enzyme-matrix complex was washed twice with 1 mL of 20 mM Tris-HCl buffer (pH 8.0) and reused for the next run. The experiments were performed in triplicate, and the data were expressed as mean values.

### 3.8. Storage Stability of the Immobilized Enzyme

The operational stability was assessed for the immobilized *Bl*GGT (~1.2 g, wet weight) during storage in 100 mL of 20 mM Tris-HCl (pH 8.0) at 4 °C. At a specific time interval, aliquots (1 mL) were withdrawn to determine the GGT activity under the standard assay conditions. The experiments were performed in triplicate, and the data were expressed as mean values.

## 4. Conclusions

In this study, magnetic iron oxide nanoparticles are successfully synthesized by the thermal co-precipitation of ferric and ferrous chloride. The coating of Fe_3_O_4_ with APES has been proven to be efficient for the covalent immobilization of *Bl*GGT. The immobilization process provides optimal recovery of activity and ideal conditions for the transpeptidation reaction. Moreover, the immobilized enzyme shows good durability and could be readily recovered by magnetic separation. To the best of our knowledge, this is the first report dealing with the covalent immobilization of a microbial GGT on surface-modified magnetic nanoparticles. The achieved stability makes the use of expensive enzymes economically viable and, therefore, opens new horizons for GGT-mediated catalysis in biotechnology.
